# Mizhuo Guanchangye enema delays the decline of renal function in rats with chronic kidney disease by intervening in the TLR4/MyD88/NF-κB pathway

**DOI:** 10.3389/fmed.2024.1454506

**Published:** 2024-10-28

**Authors:** Han Li, Peng Xu, Xiaomei Zhang, Naijing Ye, Fang Xu, Bo Liang

**Affiliations:** ^1^The Third Affiliated Hospital of Zhejiang Chinese Medical University, Hangzhou, China; ^2^The First Affiliated Hospital of Henan University of Traditional Chinese Medicine, Zhengzhou, China; ^3^Guangxi Botanical Garden of Medicinal Plant, Nanning, China; ^4^TCM Regulating Metabolic Diseases Key Laboratory of Sichuan Province, Hospital of Chengdu University of Traditional Chinese Medicine, Chengdu, China; ^5^Traditional Chinese Medicine Hospital of Meishan, Meishan, China; ^6^Department of Oncology, Tongde Hospital of Zhejiang Province, Hangzhou, China

**Keywords:** chronic kidney disease, Mizhuo Guanchangye, gut-kidney axis, gut-derived endotoxin, inflammation

## Abstract

**Background:**

Chronic kidney disease (CKD) is a prevalent chronic condition that poses a significant threat to human health. There is a close connection between the gut and kidneys, jointly influencing the onset and progression of CKD through the “gut-kidney axis.” Traditional Chinese medicine has shown potential in CKD treatment, but the specific mechanisms require further investigation.

**Objectives:**

This study aims to explore the protective effects of Mizhuo Enema (MZGCY) on kidney function in CKD rats by regulating the TLR4/MyD88/NF-κB signaling pathway.

**Methods:**

The researcher employed a CKD rat model, which was divided into four groups: Control, Model, half-dose Mizhuo Guanchangye (1/2 MZGCY), and full-dose Mizhuo Guanchangye (MZGCY). Post enema administration, assessments were conducted on kidney function indicators, which included blood urea nitrogen (BUN), serum creatinine (SCR), and 24-h urinary protein. Additionally, measurements were taken for intestinal toxic substances such as indoxyl sulfate (IS) and lipopolysaccharide (LPS), as well as inflammatory factors interleukin-6 (IL-6) and tumor necrosis factor-alpha (TNF-*α*). Examinations of pathological changes in both the intestines and kidneys were also performed. During this process, immunofluorescence was utilized to detect the expression levels of proteins toll-like receptor 4 (TLR4), myeloid differentiation primary response 88 (MyD88), and nuclear factor kappa B (NF-κB) in the intestinal tissues.

**Results:**

It was found that after enema treatment, the BUN, SCR, and 24-h urinary protein levels in the MZGCY and 1/2 MZGCY groups significantly decreased, indicating notable improvement in kidney function. Compared to the model group, the IS, LPS, IL-6, and TNF-*α* levels in the MZGCY and 1/2 MZGCY groups were significantly reduced. Immunofluorescence showed a marked decrease in the expression of TLR4, MyD88, and NF-κB proteins in the intestines of the MZGCY group.

**Conclusion:**

MZGCY significantly reduces the levels of intestinal toxins and inflammatory factors in the serum of CKD rats by interfering with the TLR4/MyD88/NF-κB signaling pathway, thereby improving intestinal and renal pathological changes and delaying CKD progression. This study demonstrates that MZGCY has significant renal protective effects, providing a new potential approach for CKD treatment.

## 1 Introduction

Chronic kidney disease (CKD) is characterized by chronic impairment of kidney structure and function caused by various factors, with evidence of kidney damage lasting more than 3 months. This includes pathological damage with both normal and abnormal kidney GFR, alterations in blood or urine components, imaging abnormalities, or an unexplained decrease in GFR (<60 mL/min·1.73 m^2^) lasting over 3 months. Epidemiological data indicate that CKD has a high prevalence worldwide, with rates varying from 5 to 13% across different regions (1–3). The long-term nature of CKD requires ongoing monitoring and treatment, and progression to end-stage renal disease necessitates renal replacement therapy, which significantly affects the daily lives of patients and their families, and places a considerable burden on public health systems (4).

The “gut-kidney axis” theory was initially derived from Ritz’s concept of “gut-kidney syndrome,” which highlights the close connection between the gut and kidneys. In 2011, Meijers et al. (5) formally introduced the “gut-kidney axis” theory at an international dialysis conference. Based on this, Pahl et al. (6) proposed the “chronic kidney disease-colon axis” concept in 2015, which elucidated the link between gut microbiota dysbiosis and renal pathophysiology, offering new perspectives for clinical practice. The core aspects of the “gut-kidney axis” theory are explained as follows: Firstly, in the later stages of chronic kidney disease, metabolic waste cannot be excreted via the gut-kidney pathway, thus accumulates in the body, potentially entering the intestinal wall through the bloodstream. Secondly, after kidney injury, the increased uremic toxins in the blood also penetrate the intestinal wall, leading to significant changes in the intestinal epithelial and microbiome environment, resulting in an increase in pathogenic bacteria and a decrease in beneficial bacteria. The entry of this disruption facilitates toxin into intestinal epithelial cells, severely damages the intestinal barrier and increases intestinal permeability. Consequently, intestinal toxins enter the bloodstream, promoting hypertension and kidney diseases (7) and accelerating CKD progression (8–11). Additionally, pathogens in the bloodstream activate the intestinal mucosal immune system, inducing systemic micro-inflammation, which subsequently leads to renal micro-inflammation and worsens chronic kidney disease (12). Xifan Wang et al. (13) discovered that, for patients with end-stage renal disease (ESRD), a dysregulated gut microbiome forms a detrimental metabolome and exacerbates clinical outcomes, suggesting that the gut microbiome could be a promising target for reducing uremic toxicity for those patients. The gut microbiota is closely associated with the impaired health-related quality of life(HRQoL) in renal transplant patients, with particularly noticeable effects on physical and mental health scores (14). Sibei Tao et al. (15) confirmed that the composition of the gut microbiota is associated with the occurrence of diabetes mellitus (DM) and diabetic nephropathy (DN), and the level of g_Prevotella_9 in feces can distinguish DM from the healthy control group (HC), while the difference between DN and DM lies in the variation of g_Escherichia-Shigella and g_Prevotella_9, which may aid in the physiological and pathological diagnosis of DM and DN. The supplementation of probiotics, specifically *Bifidobacterium bifidum* BGN4 and *Bifidobacterium longum* BORI, can alleviate intestinal inflammation in aged mice and consequently lead to an improvement in renal inflammation (16).

The present study has established that renal replacement therapy does not fully address the removal of protein-bound uremic toxins, underscoring an urgent need for new therapeutic strategies to curb uremic toxin accumulation in CKD patient (17). In this regard, nephrologists have made significant strides by embracing the “gut-kidney axis” theory, targeting the reduction of uremic toxins through strategies such as dietary control and nutritional supplementation to regulate the gut’s immune response and limit endotoxin production (18). This includes a combination of ketone analogues, essential amino acids, and a low-protein diet to manage urea levels (19), and the promotion of short-chain fatty acid production from high-resistant starch to slow the deterioration of kidney function (20). Oral adsorbents like activated charcoal and phosphate binders have been utilized to reduce uremic toxins, with probiotics and prebiotics showing particular effectiveness in clearing toxins like indoxyl sulfate and p-cresyl sulfate formed via protein binding (21). Sevelamer carbonate has emerged as a promising agent, mitigating inflammation, enhancing uremic toxin clearance, and adsorbing key uremic toxins (22). Furthermore, there is a growing interest in fecal transplantation, oral microbiota modulators for intestinal mucosal repair, and intensified dialysis techniques to improve the clearance of protein-bound uremic toxins, which conventional hemodialysis fails to eliminate (22).

Traditional Chinese medicine also offers a valuable approach, as herbal extracts contain phytochemical polyphenols and competitors for albumin binding, such as salvianolic acids from Danshen (*Salvia miltiorrhiza*), which have the potential to treat renal fibrosis in chronic kidney disease (CKD) by inhibiting EZH2 (23), sulfotransferase (SULT) inhibitors (quercetin, chlorogenic acid, curcumin, and resveratrol), showing potential in inhibiting the production of gut-derived toxins (24–28). These botanical agents not only hasten gut motility and preserve microbiota balance but also reduce renal fibrosis and protect against further renal damage (29, 30). Modern Chinese medicine practitioners, recognizing the advantages of traditional Chinese herbal treatments for CKD, have found that using herbal enemas can enhance the elimination of intestinal toxins, thereby reducing endotoxin levels and preserving residual kidney function (31, 32). A host of herbs—including rosemary, hibiscus, turmeric, red clover, emblic, pomegranate, goji berry, chili pepper, and moringa olive—display angiotensin-converting enzyme (ACE) inhibitory activity that is similar to the effects of medications like captopril and enalapril, which can lessen kidney damage (33).

The gut-kidney axis has emerged as a significant research focus in recent years, with promising new directionswith modern medical methods, including *in vivo* animal models and *in vitro* cell models, to understand the mechanisms of gut microbiota balance and the interactions between intestinal mucosal barrier function and CKD (34, 35). Lipopolysaccharide (LPS) is an inflammatory marker involved in CKD pathogenesis (15). Dysbiosis in the gut microbiota impairs the expression of tight junction proteins, resulting in increased intestinal permeability and the translocation of LPS from Gram-negative bacteria into the bloodstream (36). This is potentially associated with metabolic inflammation and CKD progression (37, 38). Research indicates that indoxyl sulfate (IS) concentrations in healthy individuals range from 0.10 to 2.39 μM, while in CKD patients, the total IS concentration exceeds 500 μM. As kidney function declines, IS causes kidney damage through the production of reactive oxygen species (ROS), damages to antioxidant systems, and increases inflammatory response, induction of fibrosis, and reduction of protective proteins (39–44).

TLR4/MyD88/NF-κB is an immune-inflammatory signaling pathway that exists in various cells and tissues throughout the human body, and is closely linked to inflammation, immunity, and infection. This study uses a CKD rat model induced by adenine suspension gavage to examine the protective effects of Mizhuo Enema (MZGCY) on both the kidneys and intestines of CKD rats. The study aims to determine whether MZGCY inhibits inflammation in CKD rats’ kidneys and intestines by regulating the TLR4/MyD88/NF-κB signaling pathway, thereby providing a novel approach for CKD treatment.

## 2 Experimental materials

### 2.1 Experimental animals

Male Sprague–Dawley rats (6–8 weeks old, 220–240 g) were procured from SPF Biotechnology Co., Ltd. (Beijing, China). This experiment has passed the ethical approval for animal welfare at Chengdu University of Traditional Chinese Medicine (batch number 2024142).

### 2.2 Experimental drugs

The Chinese herbal prescription is prepared as follows: Cimicifuga 5 g, Rhubarb 30 g, *Polygonum cuspidatum* 10 g, Scutellaria baicalensis 15 g, Artemisia capillaris 10 g, Winter melon peel 5 g, Motherwort 5 g, Leech 5 g, Earthworm 5 g, Safflower 5 g, Chicken blood vine 5 g. All herbs were purchased from the Affiliated Hospital of Chengdu University of Traditional Chinese Medicine. The preparation process is as follows: (1) Pulverization: Grind the above herbs into coarse powder using a grinder for easier decoction. (2) Decoction: First, decoct all herbs with 1,000 mL of water until about 400 mL remains, then collect the supernatant. Second, add 600 mL of water to the remaining dregs and decoct until about 200 mL remains. Third, collect the supernatant and mix the supernatants with both decoctions. (3) Concentration: Settle the mixture, filter out impurities concentrate the supernatant, add distilled water to 100 mL, sterilize for 30 min, seal, and refrigerate. The concentration of the herbal extract is 1 g/mL.

## 3 Experimental methods

### 3.1 Animal rearing

Animals were raised at the Animal Center of Chengdu University of Traditional Chinese Medicine, with free access to food and water, under closed management. They were acclimated for 1 week.

### 3.2 Animal grouping

All rats were tagged using ear marks, and were randomly assigned to four groups using SPSS software: blank group, model group, half-dose traditional Chinese medicine enema group (1/2 MZGCY), and traditional Chinese medicine enema group (MZGCY), with six rats in each group.

### 3.3 Experimental modeling

After the experiment began, except for the blank group, the other four groups were modeled for chronic kidney disease using adenine based on the Yokozawa method.

Every morning, the rats were administered under a 2.5% adenine suspension (200 mg/d/kg) (Dalian, Meilun Biotechnology Co., Ltd.)via gavage. At the end of the fourth week, two rats from each group were randomly selected for blood sampling from the medial canthus to measure serum creatinine and blood urea nitrogen, assessing the modeling status.

From the fifth week, rats under a 2.5% adenine suspension (200 mg/d/kg) was administered every other day to maintain disease progression until the seventh week, when adenine gavage was stopped. During this period, the blank group was gavaged with an equivalent volume of distilled water.

### 3.4 Experimental administration

After the experiment began, each group of rats were given the corresponding drug treatment. According to the human-animal equivalent dose ratio, the dosage for adults is 1.67 g/kg/d. Based on this, the dosage for rats was calculated to be 10.37 g/kg/d (considering the raw drug amount) (45). A straight-tip gavage needle was inserted approximately 8 cm into the rat’s rectum. The anus was pinched and the needle was fixed in place while the drug was injected slowly. The anus was then held closed for about 2 min to achieve the effect of a retained enema.

### 3.5 Specimen collection

On the last day of the eighth week, all rats were fasted for 12 h and anesthetized with an intraperitoneal injection of 3% sodium pentobarbital at a dose of 0.2 mL/100 g. Blood was collected under anesthesia, bilateral kidney and intestinal specimens were harvested. Pathological sections were prepared and stained with HE and Masson.

### 3.6 Index detection

Peripheral blood was collected from the rats, and enzyme-linked immunosorbent assay (ELISA) kits(Wuhan, Boshide Biological Engineering Co., Ltd.) were used to measure the levels of gut-derived endotoxin indoxyl sulfate (IS), lipopolysaccharide (LPS), and pro-inflammatory cytokines (TNF-*α*, IL-6) in each group(TNF-α, IL-6 were procured from Multi Science (Lianke) Biotech Co., Ltd). Blood urea nitrogen was measured using a fully automated biochemical analyzer (Shenzhen, Mindray Bio-Medical Electronics Co., Ltd., Mindray BS-420); urinary protein concentration was detected using an automatic analyzer with the bicinchoninic acid (BCA) (Beijing, Soleibao Technology Co., Ltd.) colorimetric method.

#### 3.6.1 Morphological changes under light microscopy

To examine the intestinal and renal pathological changes in CKD rats of the SSKE group, hematoxylin and eosin (H&E) (Wuhan, Google Biotechnology Co., Ltd.)staining were used, followed by observation under a light microscope (CX33, Olympus, Tokyo, Japan). Additionally, to observe the improvement in renal fibrosis in CKD model rats treated with MZGCY, the author used Masson’s trichrome staining (Wuhan, Google Biotechnology Co., Ltd.) and periodic acid-Schiff (PAS) staining according to standard protocols.

#### 3.6.2 Morphological changes under electron microscopy

Specimen fixation: Fresh tissues were sampled, minimizing mechanical damage like pulling, bruising, and compression. Tissue volume generally did not exceed 1 mm × 1 mm × 1 mm. Samples were quickly placed in electron microscope fixation solution at 4°C for 2–4 h. Dual staining with uranyl acetate and lead citrate (2% uranyl acetate saturated alcohol solution and lead citrate, each stained for 15 min) was conducted, followed by observation under a transmission electron microscope(Hitachi Company, H-7650) for image acquisition and analysis.

#### 3.6.3 Immunohistochemical staining

Paraffin sections of ileum and colon tissues were washed with PBS and fixed with 4% paraformaldehyde. Immunohistochemical staining was performed to assess the expression of occludin, tight junction protein-1 (ZO-1), DAPI (nuclear staining) [ZO-1 antibody, claudin-1 antibody, and occludin antibody were procured from Protein-tech Group, Inc. (Wuhan, China)]. After sealing, the sections were observed and imaged using a fluorescence microscope (Nikon Eclipse C1, Japan). The fluorescence intensity was measured using ImageJ software.

#### 3.6.4 Immunofluorescence staining

To confirm that traditional Chinese medicine regulates the gut-kidney axis through the NF-κB signaling pathway to improve CKD, the author performed immunofluorescence staining for verification. Paraffin sections of the ileum, colon, and kidney were deparaffinized, hydrated, and subjected to antigen retrieval. After blocking with 10% normal goat serum, the sections were incubated overnight at 4°C with primary antibodies. Nuclei were stained with DAPI. The sections were then washed with PBS, auto fluorescence quenching agent was added, and the sections were sealed with anti-fade mounting medium. After sealing, the sections were observed under a fluorescence microscope (Nikon Eclipse C1, Japan) and the corresponding images were captured.

## 4 Statistical analysis

Data were analyzed using SPSS version 22 (SPSS Inc., Chicago, IL, United States). Continuous variables were expressed as means ± standard deviations; non-parametric tests are used for non-normally distributed data. For normally distributed data, group comparisons were performed using one-way ANOVA. If variances are equal, Tukey’s HSD test was used for pairwise comparisons; if variances are unequal, the Games-Howell test was applied. Differences were considered statistically significant at *p* < 0.05.

## 5 Experimental results

### 5.1 Histopathological changes in the kidneys of each group of rats

**Figure 1:**
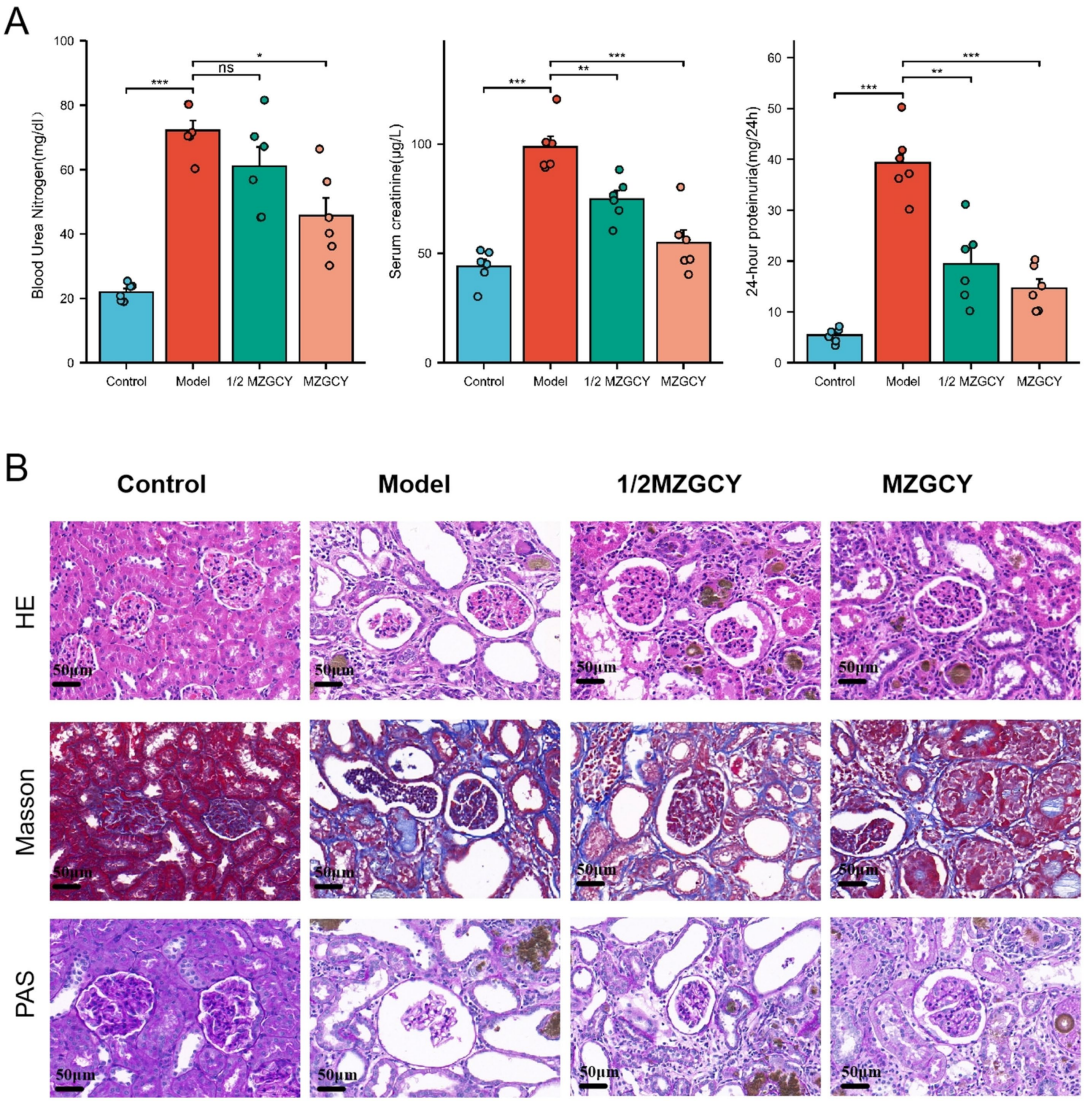
**(A)** MZGCY enema therapy can ameliorate BUN, SCR, and 24-h proteinuria in CKD rats; **(B)** illustrates the renal pathological improvements in rats following Mizhuo Guanchangye (MZGCY) treatment. The first row displays hematoxylin and eosin (H&E) staining of kidney tissues across all groups; the second row shows Masson’s trichrome staining of kidney tissues in each group; and the third row presents Periodic Acid-Schiff (PAS) staining of kidney tissues in each group.

Histological characteristics of the kidneys in the model group rats included glomerular hypertrophy, thickening of the glomerular basement membrane (GBM), mesangial matrix expansion, and vacuolar degeneration of renal tubular epithelial cells. Following treatment with MZGCY, glomerular hypertrophy, mesangial matrix expansion, and renal tubular interstitial damage were partially ameliorated. Sections stained with Masson trichrome indicated that renal fibrosis improved following MZGCY treatment. Additionally, after MZGCY enema, the accumulation of extracellular matrix (ECM) in the rat kidneys was also improved (Figure 1A).

After enema treatment, the serum urea nitrogen (BUN) levels in the Control, Model, 1/2 MZGCY, and MZGCY groups were 21.98 ± 2.66, 72.17 ± 7.48, 61.03 ± 14.59, and 45.71 ± 13.41 mg/dL, respectively; serum creatinine (SCR) levels were 44.09 ± 7.72, 98.65 ± 11.81, 74.86 ± 9.48, and 54.88 ± 14.11 μg/L; and 24-h urinary protein levels were 5.40 ± 1.42, 39.31 ± 6.70, 19.39 ± 7.66, and 14.68 ± 4.33 mg/24 h. The Shapiro–Wilk normality test showed that BUN, SCR, and 24-h proteinuria data were approximately normally distributed within each group (*p* > 0.05). Levene’s test revealed unequal variances for BUN and 24-h proteinuria data (*p* < 0.05), while variances for SCR data were equal (*p* < 0.05). Overall testing using Welch’s one-way ANOVA and multiple hypothesis testing (Games-Howell or Tukey HSD post-hoc tests) demonstrated that BUN, SCR, and 24-h proteinuria levels were significantly elevated in the Model group compared with the Control group (*p* < 0.01). Compared with the Model group, the 1/2 MZGCY group showed decreases in BUN, SCR, and 24-h proteinuria, with significant differences in SCR and 24-h proteinuria (*p* < 0.01). The full-dose MZGCY group also exhibited decreases in BUN, SCR, and 24-h proteinuria, with significant differences in BUN, SCR, and 24-h proteinuria (*p* < 0.01) (Figure 1B).

### 5.2 Morphological changes in the ileum and colon of each group of rats

**Figure 2:**
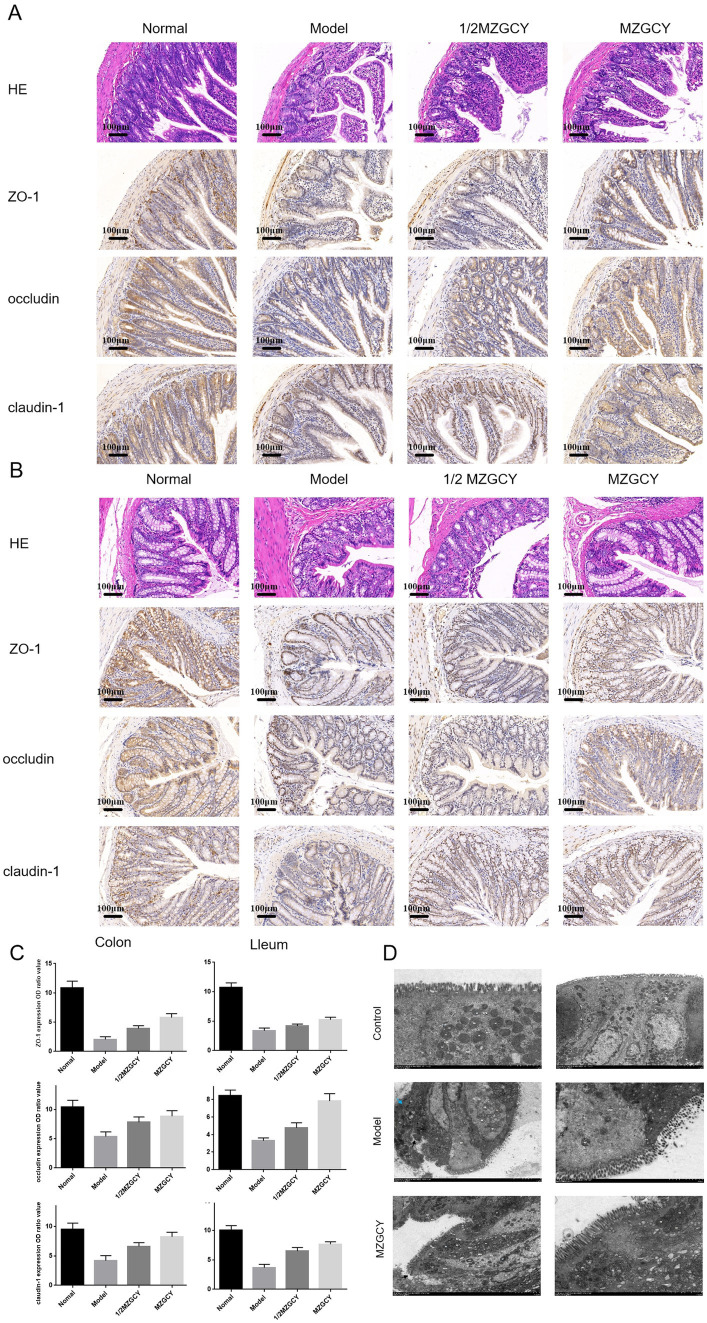
MZGCY ameliorates intestinal pathology in rats. **(A)** HE staining and immunohistochemistry of the ileal tissue in each group; **(B)** HE staining and immunohistochemistry of the colonic tissue in each group; **(C)** comparison of the expression levels (OD values) of ZO-1, Occludin-1, and Claudin-1 in the ileum and colon tissues among the four groups of rats; **(D)** are transmission electron microscopy results of the intestines in control group, model group, and MZGCY group, respectively.

The intestinal barrier is essential for maintaining intestinal function; thus, we assessed the expression of intestinal barrier markers in each group of rats. H&E staining indicated that MZGCY treatment reduced intestinal damage in model rats compared with the model group. Immunohistochemical staining demonstrated that in the normal group, ZO-1, Occludin-1, and claudin-1 proteins were evenly distributed at the top of the intestinal epithelial cells, forming a honeycomb or dot-like pattern. However, these proteins were significantly reduced in the intestines of the rats in model group. Compared with the model group, the protein levels of ZO-1, Occludin-1, and claudin-1 in the intestines of the MZGCY group were significantly restored. These results suggest that MZGCY has a protective effect on the intestinal barrier of model rats (Figures 2A–C).

Electron microscopy revealed that the intestinal barrier structure in the control group (Figure 2D) was well-maintained, with microvilli (Mv) arranged neatly and uniformly, although slightly shortened; epithelial cells showed no signs of edema, and the chromatin in the cell nucleus (N) was evenly distributed, predominantly as euchromatin; mitochondria (M) were not swollen, mostly oval-shaped, with a uniform intramembrane matrix and neatly arranged cristae; the rough endoplasmic reticulum (RER) showed no dilation, with abundant surface ribosomes. Tight junctions (TJ), adherens junctions (ZA), and desmosomes (De) were visible between cells, with clear structures, abundant tension filaments, complete complex connections, and tight connections, with no widening of the intercellular spaces observed.

Model group (Figure 2D): Moderate damage to the intestinal barrier, with microvilli (Mv) locally disarranged and detached (black arrows), varying in length; epithelial cells irregularly arranged, slightly condensed (blue arrows), with some localized mild edema; nuclear chromatin (N) evenly distributed, mainly euchromatin; numerous mitochondria (M), slightly swollen, increased in size, cristae fractured and shortened, matrix pale and uneven; partial mild dilation of rough endoplasmic reticulum (RER), surface ribosomes locally lost; tight junctions (TJ), adherens junctions (ZA), desmosomes (De)were observed between cells, with narrow intercellular spaces, compound connections abundant in tension filaments, tightly connected, mostly intact without obvious fractures or disappearance.

MZGCY group (Figure 2D): Mild damage to the intestinal barrier, with densely arranged microvilli (Mv); some epithelial cells mildly edematous, nuclear chromatin (N) evenly distributed, mainly euchromatin with some heterochromatin marginated; mitochondria (M) mildly swollen, increased in size, cristae partially fractured and disorganized, matrix pale and uneven; rough endoplasmic reticulum (RER) mildly to moderately dilated, surface ribosomes lost; tight junctions (TJ), adherens junctions (ZA), desmosomes (De) observed between cells, with narrow intercellular spaces, compound connections abundant in tension filaments, tightly connected, with some local loss of intercellular connections (black arrows).

**Figure 3:**
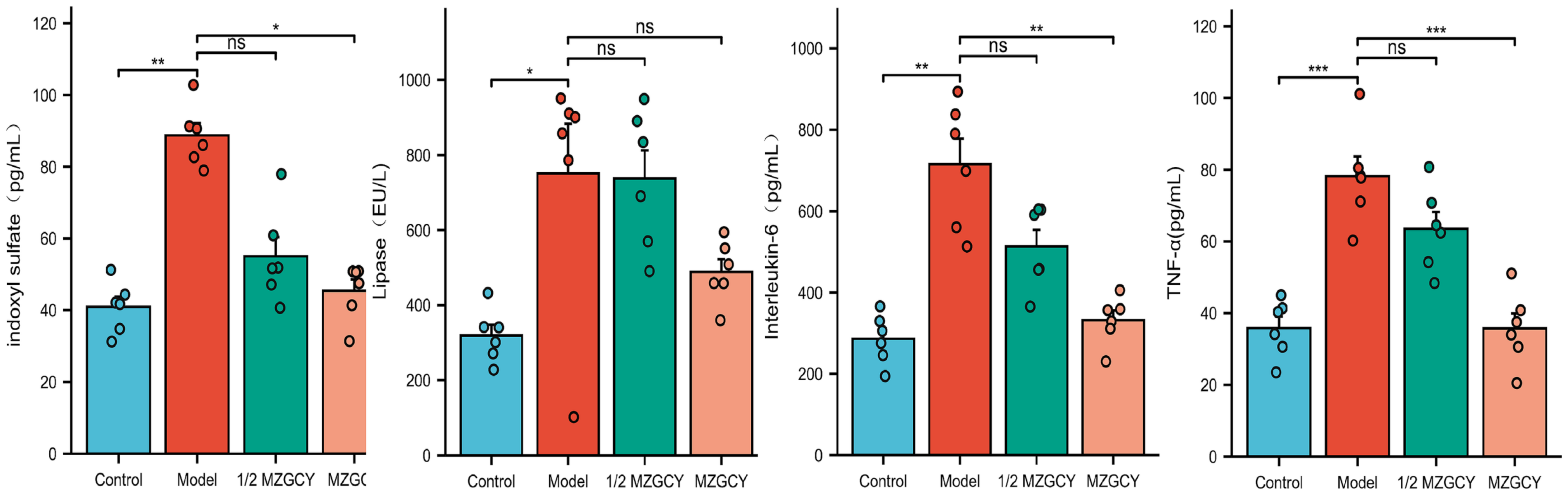
Using MZGCY enema treatment can decrease the levels of intestinal toxins and inflammatory factors in the serum of CKD rats.

In order to explore the mechanism of renal protection of CKD by traditional Chinese medicine through the renal-gut axis, we detected the levels of intestinal toxic substances (IS and LPS) and inflammatory factors (IL-6 and TNF-*α*) in the serum of rats in the Control group, Model group, 1/2 MZGCY group, and MZGCY group after enema administration(Figure 3). The IS levels were found to be 40.91 ± 7.09, 88.73 ± 8.34, 55.03 ± 13.02, 45.43 ± 7.79 pg./mL for the four groups, respectively, while the LPS levels were 319.22 ± 70.29, 751.21 ± 323.06, 737.40 ± 183.76, 488.56 ± 82.27 EU/L, and the IL-6 levels were 286.13 ± 61.33, 715.66 ± 153.41, 512.99 ± 100.68, 331.92 ± 59.14 pg./mL, with TNF-*α* levels at 35.82 ± 7.96, 78.18 ± 13.44, 63.51 ± 11.54, 35.75 ± 10.25 pg./mL, respectively. Shapiro–Wilk normality test revealed that the IS data from the MZGCY group and the LPS data from the Model group did not conform to a normal distribution (*p* < 0.05), while IL-6 and TNF-*α* data approximated a normal distribution within each group (*p* > 0.05). Additionally, Levene’s test indicated that the variances of the observed variables in the IL-6 data were not equal across groups (*p* < 0.05), whereas the variances in the IS, LPS, and TNF-*α* data were equal (*p* > 0.05). Ultimately, the overall test (Kruskal-Wallis Test or Welch one-way ANOVA test) and multiple comparison test (Games-Howell *post hoc* test or Tukey HSD *post hoc* test) analyses indicated that compared to the control group, the levels of IS, LPS, IL-6, and TNF-*α* in the model group were significantly elevated (*p* < 0.01). Compared to the model group, the 1/2 MZGCY group showed a decrease in IS, LPS, IL-6, and TNF-α, with significant statistical differences in IS (*p* < 0.01) and statistical differences in IL-6 (*p* < 0.05). Compared with the model group, the MZGCY group showed a decrease in IS, LPS, IL-6, and TNF-α, with significant statistical differences observed in IS, IL-6, and TNF-α (*p* < 0.01).

**Figure 4:**
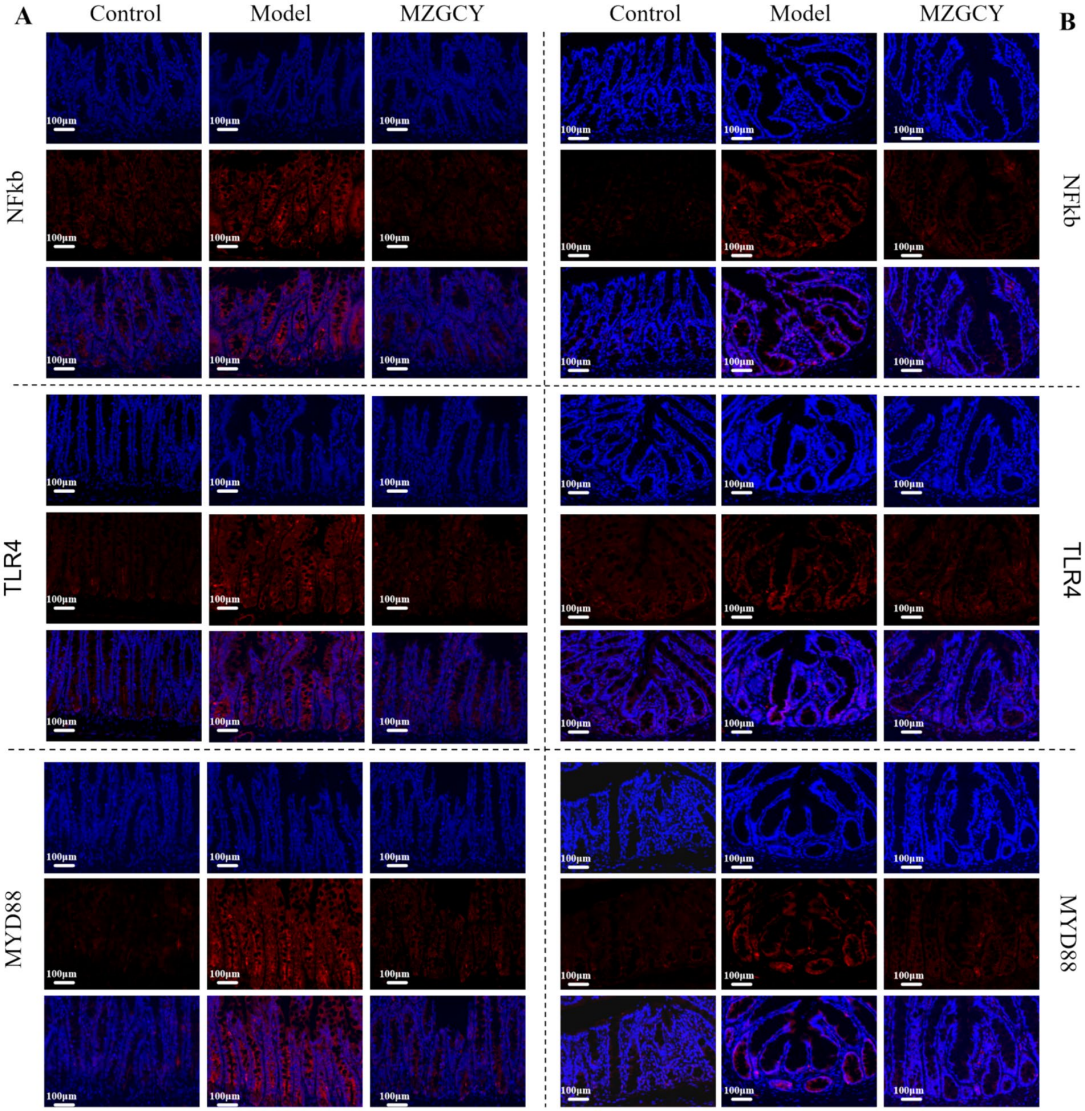
Immunofluorescence of intestinal tissues in CKD rats from control, model, and MZGCY groups. **(A)** Immunofluorescence of ileum tissues in each group; **(B)** immunofluorescence of colon tissues in each group.

We employed immunofluorescence techniques to further assess the expression of TLR4, MyD88, and NF-κB proteins in the intestines of each group(Figure 4). Immunofluorescence images showed that the expression of TLR4, MyD88, and NF-κB proteins was significantly increased in the intestinal tissues of the model group rats compared to the normal control group; compared to the model group, the MZGCY group rats had significantly lower levels of TLR4, MyD88, and NF-κB in their intestinal tissues.

## 6 Discussion

Traditional Chinese medicine is frequently used to treat CKD. A multicenter, randomized, double-blind clinical trial focused on the recurrence treatment of CKD stages 3–4 indicated that traditional Chinese medicine contributes to the delay in serum creatinine increase and the reduction in glomerular filtration rate, providing long-term benefits for patients with moderate to severe renal impairment (46, 47). Research on animals indicates that TCM can decrease the levels of 24-h urinary protein, creatinine, and urea nitrogen in rats suffering from chronic renal failure, thereby aiding in the postponement of renal failure progression. This effect is associated with the reduction of renal tubular injury and the suppression of apoptosis in renal tubular epithelial cells (48).

Studies indicate that dysbiosis of the gut microbiota, impairment of the intestinal epithelial barrier, and dysfunction of immune cells are prominent characteristics of the uremic environment, which exacerbates the inflammatory status in CKD (49). Suppression of inflammatory factor release and the expression of inflammation-related pathways may halt or slow the progression of CKD (50, 51). TLR4, an important immune receptor mediating inflammation, plays a critical role in the inflammatory response associated with the progression of CKD (52, 53). TLR4 can work in conjunction with TLR2, influencing the regulation of gut microbiota and the preservation of barrier integrity, which is especially pertinent to the intestinal barrier. The expression levels of TLR2 and TLR4 in intestinal epithelial cells (IEC) are depended on the symbiotic gut microbiota (54). The stimulation of IEC *in vitro* with lipopolysaccharide (LPS) increases the expression of TLR2 and TLR4 (55). LPS can also be recognized by TLR2 and TLR4 receptors, leading to the activation of the MyD88/NF-κB pathway, which enhances the expression of various inflammatory factors, decreases the synthesis and distribution of ZO-1, and disrupts intestinal tight junctions (TJs) (56). When TLR4 enhances inflammatory and anti-apoptotic signals, the key transcription factor involved is NF-κB, leading to adverse effects such as tumor formation (57). The signaling pathways was mediated by TLR4 consisting primarily of MyD88-dependent and MyD88-independent pathways (58). The MyD88-dependent pathway is considered the classical pathway, wherein TLR4 interacts with the downstream MyD88 adaptor protein, leading to the phosphorylation of the inhibitor of NF-κB (IκB) (44). This phosphorylation of IκB activates NF-κB, allowing it to move from the cytoplasm to the nucleus, where it facilitates the release of inflammatory factors such as IL-6 and TNF-*α* (59, 60). TLR4 and MyD88 proteins are expressed in the cytoplasm of renal tubular epithelial cells and glomerular parenchymal cells, while NF-κB is expressed in both the cytoplasm and nucleus of these cells. Additionally, inhibition of the TLR4/MyD88/NF-κB pathway can reduce inflammatory responses in glomerular cells and extracellular matrix accumulation (61). Thus, the TLR4/MyD88/NF-κB pathway is closely related to the development of CKD, and regulating this pathway may mitigate CKD progression. In this study, TLR4, MyD88, and NF-κB protein expression in the intestinal tissue of CKD rats was found to be higher than that in normal model rats. Furthermore, CKD rats exhibited elevated serum levels of IL-6 and TNF-*α* compared with the normal control group, indicating that the TLR4/MyD88/NF-κB signaling pathway may be activated in these model rats, promoting the inflammatory response. However, this study did not explore the specific changes in gut microbiota species and genera in CKD rats and their impacts on intestinal barrier function, nor did it clarify which TLR4 heterodimer is activated by Peach Kernel Decoction to inhibit the TLRs/NF-κB signaling pathway under the experimental conditions.

According to the principles of traditional Chinese medicine formulation theory, MZGCY is thought to possess functions such as dispelling wind (Chantui, Siler), draining turbidity (Rhubarb, Huzhang), removing dampness (Scutellaria, Artemisia), and promoting circulation (Leech, Earthworm), with its efficacy closely related to its active components. Several traditional Chinese medicines have been studied for their application in CKD and other renal diseases. Oroxylin A (OA), a key active component of Scutellaria, has been shown to alleviate mitochondrial injury by inducing the expression of BNIP3 (62). Studies report that the absence of BNIP3 exacerbates acute kidney injury (AKI) induced by ischemia–reperfusion (IR), while over expression of BNIP3 not only reverses the decline of mitochondrial autophagy, but also reduces kidney damage, indicating that BNIP3-mediated mitochondrial autophagy plays a protective role in AKI (63, 64). The electron microscopy results in this study demonstrated that mitochondria in the model group of rats were swollen, enlarged, with fragmented and shortened cristae, and a pale or uneven matrix. MZGCY was found to improve mitochondrial edema and alleviate mitochondrial damage, possibly associated with the effects of Oroxylin A. Rhein, the primary active component of Rhubarb, can significantly enhance renal function and reverse markers of renal fibrosis such as FN1, collagen *α*I, α-SMA, NF-κB, and NKD2, effectively inhibiting the production of TNF-α, IL-6, and IL-1β, while suppressing NF-κB activation and NKD2 expression (65), aligning with the findings of this study. Hirudin, the main active ingredient from leeches, was studied by Chunli Long et al. (66), who suggested that high doses of hirudin can downregulate levels of indoxyl sulfate (IS), p-cresol (PCS), ammonia (NH3), and LPS in CKD rats. Treatment with high doses of hirudin significantly ameliorated renal and colonic damage in CKD rats, reversing the expression of relevant factors and proteins, while regulating the homeostasis of gut microbiota at the same time. This suggests that hirudin may correct the imbalance of the “gut-kidney axis” in CKD patients, restore intestinal epithelial barrier function, and postpone the decline in renal function. The results from Chunli Long et al. are consistent with this study, but given that the dosage of hirudin used here was not high. It is possible that the synergistic effects of the various traditional Chinese medicines employed may contribute, although the specific mechanisms of synergy warrant further investigation. Therefore, it can be inferred that MZGCY may enhance renal function through multiple mechanisms.

The colon plays a significant role in generating uremic toxins, and the application of traditional Chinese medicine via enema directly targets the colon, which can enhance gastrointestinal motility, improve intestinal barrier function, regulate intestinal microbiota, and reduce the production and absorption of intestinal-derived uremic toxins such as indole sulfate. This ultimately contributes to decreased renal fibrosis and slows the progression of kidney disease (24). This study also demonstrates that the enema of MZGCY can improve intestinal barrier function, rectify dysbiosis, inhibit systemic inflammation, and relieve renal fibrosis. The mechanism behind these effects is significantly associated with the intervention’s ability to alter gut microbiota, intestinal barrier markers, TLR4/MyD88/NF-κB inflammatory responses, and systemic inflammation.

In general, TLR4/MyD88/NF-κB is critically involved in the pathogenesis and progression of CKD. Following MZGCY intervention in CKD rats, there was a marked decrease in IL-6, TNF-*α*, and the levels of TLR4, MyD88, and NF-κB in the intestinal tissue, indicating that MZGCY may ameliorate renal damage in CKD rats, most likely to related to its ability to downregulate the TLR4/MyD88/NF-κB signaling pathway, thereby inhibiting inflammatory responses and exerting a protective effect against CKD. Nonetheless, this study’s investigation of mechanisms is still insufficient, lacking validation in cellular models, and will be further refined in future research.

## Data Availability

The raw data supporting the conclusions of this article will be made available by the authors, without undue reservation.

## References

[1] De Nicola L Minutolo R. Worldwide growing epidemic of CKD: fact or fiction? Kidney Int. (2016) 90:482–4. doi: 10.1016/j.kint.2016.05.001 27521111

[2] Kalantar-Zadeh K Jafar TH Nitsch D Neuen BL Perkovic V. Chronic kidney disease. Lancet. (2021) 398:786–802. doi: 10.1016/S0140-6736(21)00519-5 34175022

[3] Wildhaber D. 10 years' experience in ambulatory and hospital consultation in oncology at the Monthey hospital. Rev Med Suisse Romande. (1988) 108:941–7. PMID: 3206048

[4] Huang R Fu P Ma L. Kidney fibrosis: from mechanisms to therapeutic medicines. Signal Transduct Target Ther. (2023) 8:129. doi: 10.1038/s41392-023-01379-7, PMID: 36932062 PMC10023808

[5] Meijers BK Evenepoel P. The gut-kidney axis: indoxyl sulfate, p-cresyl sulfate and CKD progression. Nephrol Dial Transplant. (2011) 26:759–61. doi: 10.1093/ndt/gfq818, PMID: 21343587

[6] Pahl MV Vaziri ND. The chronic kidney disease – colonic Axis. Semin Dial. (2015) 28:459–63. doi: 10.1111/sdi.12381, PMID: 25855516

[7] Yang T Richards EM Pepine CJ Raizada MK. The gut microbiota and the brain-gut-kidney axis in hypertension and chronic kidney disease. Nat Rev Nephrol. (2018) 14:442–56. doi: 10.1038/s41581-018-0018-2, PMID: 29760448 PMC6385605

[8] Vaziri ND Wong J Pahl M Piceno YM Yuan J DeSantis TZ. Chronic kidney disease alters intestinal microbial flora. Kidney Int. (2013) 83:308–15. doi: 10.1038/ki.2012.345, PMID: 22992469

[9] Vanholder R Glorieux G. The intestine and the kidneys: a bad marriage can be hazardous. Clin Kidney J. (2015) 8:168–79. doi: 10.1093/ckj/sfv004, PMID: 25815173 PMC4370304

[10] Sommer F Anderson JM Bharti R Raes J Rosenstiel P. The resilience of the intestinal microbiota influences health and disease. Nat Rev Microbiol. (2017) 15:630–8. doi: 10.1038/nrmicro.2017.58 28626231

[11] Hugenholtz F de Vos WM. Mouse models for human intestinal microbiota research: a critical evaluation. Cell Mol Life Sci. (2018) 75:149–60. doi: 10.1007/s00018-017-2693-8, PMID: 29124307 PMC5752736

[12] Miao H Wang YN Yu XY Zou L Guo Y Su W. Lactobacillus species ameliorate membranous nephropathy through inhibiting the aryl hydrocarbon receptor pathway via tryptophan-produced indole metabolites. Br J Pharmacol. (2024) 181:162–79. doi: 10.1111/bph.16219, PMID: 37594378

[13] Wang X Yang S Li S Zhao L Hao Y Qin J. Aberrant gut microbiota alters host metabolome and impacts renal failure in humans and rodents. Gut. (2020) 69:2131–42. doi: 10.1136/gutjnl-2019-319766, PMID: 32241904 PMC7677483

[14] Swarte JC Knobbe TJ Björk JR Gacesa R Nieuwenhuis LM Zhang S. Health-related quality of life is linked to the gut microbiome in kidney transplant recipients. Nat Commun. (2023) 14:7968. doi: 10.1038/s41467-023-43431-8, PMID: 38042820 PMC10693618

[15] Tao S Li L Li L Liu Y Ren Q Shi M. Understanding the gut-kidney axis among biopsy-proven diabetic nephropathy, type 2 diabetes mellitus and healthy controls: an analysis of the gut microbiota composition. Acta Diabetol. (2019) 56:581–92. doi: 10.1007/s00592-019-01316-7, PMID: 30888537

[16] Kim MG Cho WY Chung SM Choi YE Fang Y Park MS. Altered gut microbiome plays an important role in AKI to CKD transition in aged mice. Front Med (Lausanne). (2023) 10:1238960. doi: 10.3389/fmed.2023.1238960, PMID: 38020091 PMC10644820

[17] Krukowski H Valkenburg S Madella AM Garssen J van Bergenhenegouwen J Overbeek SA. Gut microbiome studies in CKD: opportunities, pitfalls and therapeutic potential. Nat Rev Nephrol. (2023) 19:87–101. doi: 10.1038/s41581-022-00647-z 36357577

[18] Montemurno E Cosola C Dalfino G Daidone G de Angelis M Gobbetti M. What would you like to eat, Mr CKD microbiota? A Mediterranean diet, please! Kidney Blood Press Res. (2014) 39:114–23. doi: 10.1159/000355785, PMID: 25117687

[19] Marzocco S Dal Piaz F di Micco L Torraca S Sirico ML Tartaglia D. Very low protein diet reduces indoxyl sulfate levels in chronic kidney disease. Blood Purif. (2013) 35:196–201. doi: 10.1159/000346628, PMID: 23485887

[20] Vaziri ND Liu SM Lau WL Khazaeli M Nazertehrani S Farzaneh SH. High amylose resistant starch diet ameliorates oxidative stress, inflammation, and progression of chronic kidney disease. PLoS One. (2014) 9:e114881. doi: 10.1371/journal.pone.0114881, PMID: 25490712 PMC4260945

[21] Chen JH Chao CT Huang JW Hung KY Liu SH Tarng DC. Early elimination of uremic toxin ameliorates AKI-to-CKD transition. Clin Sci (Lond). (2021) 135:2643–58. doi: 10.1042/CS20210858, PMID: 34796904

[22] Lenglet A Fabresse N Taupin M Gomila C Liabeuf S Kamel S. Does the Administration of Sevelamer or nicotinamide modify uremic toxins or Endotoxemia in chronic hemodialysis patients? Drugs. (2019) 79:855–62. doi: 10.1007/s40265-019-01118-9 31062264

[23] Li J Wang Y Xu X Cao W Shen Z Wang N. Improved dialysis removal of protein-bound uremic toxins by salvianolic acids. Phytomedicine. (2019) 57:166–73. doi: 10.1016/j.phymed.2018.12.018, PMID: 30772752

[24] Zou C Lu ZY Wu YC Yang LH Su GB Jie XN. Colon may provide new therapeutic targets for treatment of chronic kidney disease with Chinese medicine. Chin J Integr Med. (2013) 19:86–91. doi: 10.1007/s11655-013-1351-8, PMID: 23371456

[25] Kusumoto M Kamobayashi H Sato D Komori M Yoshimura M Hamada A. Alleviation of cisplatin-induced acute kidney injury using phytochemical polyphenols is accompanied by reduced accumulation of indoxyl sulfate in rats. Clin Exp Nephrol. (2011) 15:820–30. doi: 10.1007/s10157-011-0524-z, PMID: 21858734

[26] Saito H Yoshimura M Saigo C Komori M Nomura Y Yamamoto Y. Hepatic sulfotransferase as a Nephropreventing target by suppression of the uremic toxin Indoxyl sulfate accumulation in ischemic acute kidney injury. Toxicol Sci. (2014) 141:206–17. doi: 10.1093/toxsci/kfu119, PMID: 24958931 PMC4833106

[27] Ye N Zhao P Ayue S Qi S Ye Y He H. Folic acid-modified lactoferrin nanoparticles coated with a laminarin layer loaded curcumin with dual-targeting for ulcerative colitis treatment. Int J Biol Macromol. (2023) 232:123229. doi: 10.1016/j.ijbiomac.2023.123229, PMID: 36642354

[28] Balkrishna A Sinha S Kumar A Arya V Gautam AK Valis M. Sepsis-mediated renal dysfunction: pathophysiology, biomarkers and role of phytoconstituents in its management. Biomed Pharmacother. (2023) 165:115183. doi: 10.1016/j.biopha.2023.115183, PMID: 37487442

[29] Zheng L Luo M Zhou H Chen J. Natural products from plants and microorganisms: novel therapeutics for chronic kidney disease via gut microbiota regulation. Front Pharmacol. (2022) 13:1068613. doi: 10.3389/fphar.2022.1068613 36733377 PMC9887141

[30] du J Yang M Zhang Z Cao B Wang Z Han J. The modulation of gut microbiota by herbal medicine to alleviate diabetic kidney disease – a review. Front Pharmacol. (2022) 13:1032208. doi: 10.3389/fphar.2022.1032208, PMID: 36452235 PMC9702521

[31] Peng Y Zeng Y Zheng T Xie X Wu J Fu L. Effects of Tiaopi Xiezhuo decoction on constipation and gut dysbiosis in patients with peritoneal dialysis. Pharm Biol. (2023) 61:531–40. doi: 10.1080/13880209.2023.2193595, PMID: 36994999 PMC10064829

[32] Wang F Liu C Ren LZ Li YY Yang HM Yu Y. Sanziguben polysaccharides improve diabetic nephropathy in mice by regulating gut microbiota to inhibit the TLR4/NF-kappaB/NLRP3 signalling pathway. Pharm Biol. (2023) 61:427–36. doi: 10.1080/13880209.2023.2174145, PMID: 36772833 PMC9930838

[33] Khaled A Ahmed E Mamdouh M Saad H Mohamed A Sobhy M. Natural angiotensin converting enzyme inhibitors: a safeguard against hypertension, respiratory distress syndrome, and chronic kidney diseases. Phytother Res. (2023) 37:5464–72. doi: 10.1002/ptr.7987, PMID: 37675925

[34] Crespo-Salgado J Vehaskari VM Stewart T Ferris M Zhang Q Wang G. Intestinal microbiota in pediatric patients with end stage renal disease: a Midwest pediatric nephrology consortium study. Microbiome. (2016) 4:50. doi: 10.1186/s40168-016-0195-9, PMID: 27640125 PMC5027112

[35] Nallu A Sharma S Ramezani A Muralidharan J Raj D. Gut microbiome in chronic kidney disease: challenges and opportunities. Transl Res. (2017) 179:24–37. doi: 10.1016/j.trsl.2016.04.007, PMID: 27187743 PMC5086447

[36] Cani PD Delzenne NM. The role of the gut microbiota in energy metabolism and metabolic disease. Curr Pharm Des. (2009) 15:1546–58. doi: 10.2174/138161209788168164 19442172

[37] Feng Y Weng H Ling L Zeng T Zhang Y Chen D. Modulating the gut microbiota and inflammation is involved in the effect of Bupleurum polysaccharides against diabetic nephropathy in mice. Int J Biol Macromol. (2019) 132:1001–11. doi: 10.1016/j.ijbiomac.2019.03.242 30946910

[38] Zhao T Zhang HJ Yin X Zhao HL Ma L Yan MH. Tangshen formula modulates gut microbiota and reduces gut-derived toxins in diabetic nephropathy rats. Biomed Pharmacother. (2020) 129:110325. doi: 10.1016/j.biopha.2020.110325, PMID: 32535383

[39] Lim YJ Sidor NA Tonial NC Che A Urquhart BL. Uremic toxins in the progression of chronic kidney disease and cardiovascular disease: mechanisms and therapeutic targets. Toxins (Basel). (2021) 13:142. doi: 10.3390/toxins13020142, PMID: 33668632 PMC7917723

[40] Falconi CA Junho CVC Fogaça-Ruiz F Vernier ICS da Cunha RS Stinghen AEM. Uremic toxins: An alarming danger concerning the cardiovascular system. Front Physiol. (2021) 12:686249. doi: 10.3389/fphys.2021.686249, PMID: 34054588 PMC8160254

[41] Cheng TH Ma MC Liao MT Zheng CM Lu KC Liao CH. Indoxyl sulfate, a tubular toxin, contributes to the development of chronic kidney disease. Toxins (Basel). (2020) 12:684. doi: 10.3390/toxins12110684, PMID: 33138205 PMC7693919

[42] Chang LC Sun HL Tsai CH Kuo CW Liu KL Lii CK. 1,25(OH)(2) D(3) attenuates indoxyl sulfate-induced epithelial-to-mesenchymal cell transition via inactivation of PI3K/Akt/β-catenin signaling in renal tubular epithelial cells. Nutrition. (2020) 69:110554. doi: 10.1016/j.nut.2019.110554, PMID: 31536856

[43] Yu Y Guan X Nie L Liu Y He T Xiong J. DNA hypermethylation of sFRP5 contributes to indoxyl sulfate-induced renal fibrosis. J Mol Med (Berl). (2017) 95:601–13. doi: 10.1007/s00109-017-1538-0, PMID: 28508124

[44] Neyra JA Hu MC Moe OW. Klotho in clinical nephrology: diagnostic and therapeutic implications. Clin J Am Soc Nephrol. (2020) 16:162–76. doi: 10.2215/CJN.02840320, PMID: 32699047 PMC7792642

[45] Nair AB Jacob S. A simple practice guide for dose conversion between animals and human. J Basic Clin Pharm. (2016) 7:27–31. doi: 10.4103/0976-0105.177703, PMID: 27057123 PMC4804402

[46] Zheng Y Wang NS Liu YN He LQ Jian GH Liu XS. Effects of Niaoduqing particles () on delaying progression of renal dysfunction: a Post-trial, open-label, follow-up study. Chin J Integr Med. (2019) 25:168–74. doi: 10.1007/s11655-018-2998-y 30467695

[47] Zheng Y Cai GY He LQ Lin HL Cheng XH Wang NS. Efficacy and safety of Niaoduqing particles for delaying moderate-to-severe renal dysfunction: a randomized, double-blind, placebo-controlled, multicenter clinical study. Chin Med J. (2017) 130:2402–9. doi: 10.4103/0366-6999.216407, PMID: 29052559 PMC5684630

[48] Xu YF Ruan SW Lin JM Zhang Z. Yishen Jiangzhuo granules affect tubulointerstitial fibrosis via a mitochondrion-mediated apoptotic pathway. Chin J Integr Med. (2015) 21:928–37. doi: 10.1007/s11655-015-2078-5, PMID: 25956968

[49] Evenepoel P Stenvinkel P Shanahan C Pacifici R. Inflammation and gut dysbiosis as drivers of CKD-MBD. Nat Rev Nephrol. (2023) 19:646–57. doi: 10.1038/s41581-023-00736-7, PMID: 37488276

[50] Ni Z Guo L Liu F Olatunji OJ Yin M. Allium tuberosum alleviates diabetic nephropathy by supressing hyperglycemia-induced oxidative stress and inflammation in high fat diet/streptozotocin treated rats. Biomed Pharmacother. (2019) 112:108678. doi: 10.1016/j.biopha.2019.108678, PMID: 30784905

[51] Li M Guo Q Cai H Wang H Ma Z Zhang X. miR-218 regulates diabetic nephropathy via targeting IKK-β and modulating NK-κB-mediated inflammation. J Cell Physiol. (2020) 235:3362–71. doi: 10.1002/jcp.29224, PMID: 31549412 PMC13482667

[52] Lu S Zhang H Wei X Huang X Chen L Jiang L. 2-dodecyl-6-methoxycyclohexa-2,5-diene-1,4-dione isolated from Averrhoa carambola L. root ameliorates diabetic nephropathy by inhibiting the TLR4/MyD88/NF-κB pathway. Diabetes Metab Syndr Obes. (2019) 12:1355–63. doi: 10.2147/DMSO.S209436, PMID: 31496773 PMC6689538

[53] Zhang H Lu S Chen L Huang X Jiang L Li Y. 2-Dodecyl-6-methoxycyclohexa-2,5-diene-1,4-dione, isolated from the root of Averrhoa carambola L., protects against diabetic kidney disease by inhibiting TLR4/TGFβ signaling pathway. Int Immunopharmacol. (2020) 80:106120. doi: 10.1016/j.intimp.2019.106120, PMID: 31972423

[54] Inoue R Yajima T Tsukahara T. Expression of TLR2 and TLR4 in murine small intestine during postnatal development. Biosci Biotechnol Biochem. (2017) 81:350–8. doi: 10.1080/09168451.2016.1254534, PMID: 27838962

[55] Yang X Gao XC Liu J Ren HY. Effect of EPEC endotoxin and bifidobacteria on intestinal barrier function through modulation of toll-like receptor 2 and toll-like receptor 4 expression in intestinal epithelial cell-18. World J Gastroenterol. (2017) 23:4744–51. doi: 10.3748/wjg.v23.i26.4744, PMID: 28765695 PMC5514639

[56] Liu C Jing K. Effects of toll-like receptor blockers on intestinal mucosal injury in mice with endotoxemia. Zhongguo Dang Dai Er Ke Za Zhi. (2018) 20:158–63. doi: 10.7499/j.issn.1008-8830.2018.02.015, PMID: 29429467 PMC7389237

[57] Pradere JP Dapito DH Schwabe RF. The Yin and Yang of toll-like receptors in cancer. Oncogene. (2014) 33:3485–95. doi: 10.1038/onc.2013.302, PMID: 23934186 PMC4059777

[58] Liu Y Yin H Zhao M Lu Q. TLR2 and TLR4 in autoimmune diseases: a comprehensive review. Clin Rev Allergy Immunol. (2014) 47:136–47. doi: 10.1007/s12016-013-8402-y, PMID: 24352680

[59] Tang J Xu L Zeng Y Gong F. Effect of gut microbiota on LPS-induced acute lung injury by regulating the TLR4/NF-kB signaling pathway. Int Immunopharmacol. (2021) 91:107272. doi: 10.1016/j.intimp.2020.107272, PMID: 33360370

[60] Xu GR Zhang C Yang HX Sun JH Zhang Y Yao TT. Modified citrus pectin ameliorates myocardial fibrosis and inflammation via suppressing galectin-3 and TLR4/MyD88/NF-κB signaling pathway. Biomed Pharmacother. (2020) 126:110071. doi: 10.1016/j.biopha.2020.110071, PMID: 32172066

[61] Chen F Zhu X Sun Z Ma Y. Astilbin inhibits high glucose-induced inflammation and extracellular matrix accumulation by suppressing the TLR4/MyD88/NF-κB pathway in rat glomerular mesangial cells. Front Pharmacol. (2018) 9:1187. doi: 10.3389/fphar.2018.01187, PMID: 30459606 PMC6232904

[62] Yao M Qin S Xiong J Xin W Guan X Gong S. Oroxylin a ameliorates AKI-to-CKD transition through maintaining PPARα-BNIP3 signaling-mediated mitochondrial homeostasis. Front Pharmacol. (2022) 13:935937. doi: 10.3389/fphar.2022.935937, PMID: 36081929 PMC9445212

[63] Fu ZJ Wang ZY Xu L Chen XH Li XX Liao WT. HIF-1α-BNIP3-mediated mitophagy in tubular cells protects against renal ischemia/reperfusion injury. Redox Biol. (2020) 36:101671. doi: 10.1016/j.redox.2020.101671, PMID: 32829253 PMC7452120

[64] Tang C Han H Liu Z Liu Y Yin L Cai J. Activation of BNIP3-mediated mitophagy protects against renal ischemia-reperfusion injury. Cell Death Dis. (2019) 10:677. doi: 10.1038/s41419-019-1899-0, PMID: 31515472 PMC6742651

[65] Gu M Zhou Y Liao N Wei Q Bai Z Bao N. Chrysophanol, a main anthraquinone from Rheum palmatum L. (rhubarb), protects against renal fibrosis by suppressing NKD2/NF-κB pathway. Phytomedicine. (2022) 105:154381. doi: 10.1016/j.phymed.2022.154381, PMID: 35988461

[66] Long C Zhang C Xie Y. Study on the mechanism of hirudin multi target delaying renal function decline in chronic kidney disease based on the "gut-kidney axis" theory. Naunyn Schmiedeberg's Arch Pharmacol. (2024) 397:7951–62. doi: 10.1007/s00210-023-02888-6, PMID: 38758227 PMC11450085

